# Corrigendum: Single cell RNA sequencing analysis of mouse cochlear supporting cell transcriptomes with activated ERBB2 receptor indicates a cell-specific response that promotes CD44 activation

**DOI:** 10.3389/fncel.2024.1428589

**Published:** 2024-05-24

**Authors:** Dorota Piekna-Przybylska, Daxiang Na, Jingyuan Zhang, Cameron Baker, John M. Ashton, Patricia M. White

**Affiliations:** ^1^Department of Neuroscience, University of Rochester School of Medicine and Dentistry, Rochester, NY, United States; ^2^Department of Biology, University of Rochester, Rochester, NY, United States; ^3^Genomic Research Center, University of Rochester School of Medicine and Dentistry, Rochester, NY, United States

**Keywords:** cochlear regeneration, candidate pathway, ERBB2, single cell sequencing, SIBLING, CD44

In the published article, there was an error in the **Funding** statement. This statement was erroneously written as “This work was funded by the U.S. Army Medical Research Mechanism W81XWH2010515 grant number RH190035, and NIDCD grants R01 DC014261 and R01 DC018660.”, where the inclusion of R01 DC018660 was incorrect. The correct **Funding** statement appears below.

