# Supplementary material for: Single cell RNA sequencing analysis of mouse cochlear supporting cell transcriptomes with activated ERBB2 receptor indicates a cell-specific response that promotes CD44 activation
Source: Front Cell Neurosci. 2023 Jan 6;16:1096872. doi: 10.3389/fncel.2022.1096872 (PMC9853549; doi:10.3389/fncel.2022.1096872)
Supplement: Supplementary file 1 [file Data_Sheet_1.pdf]

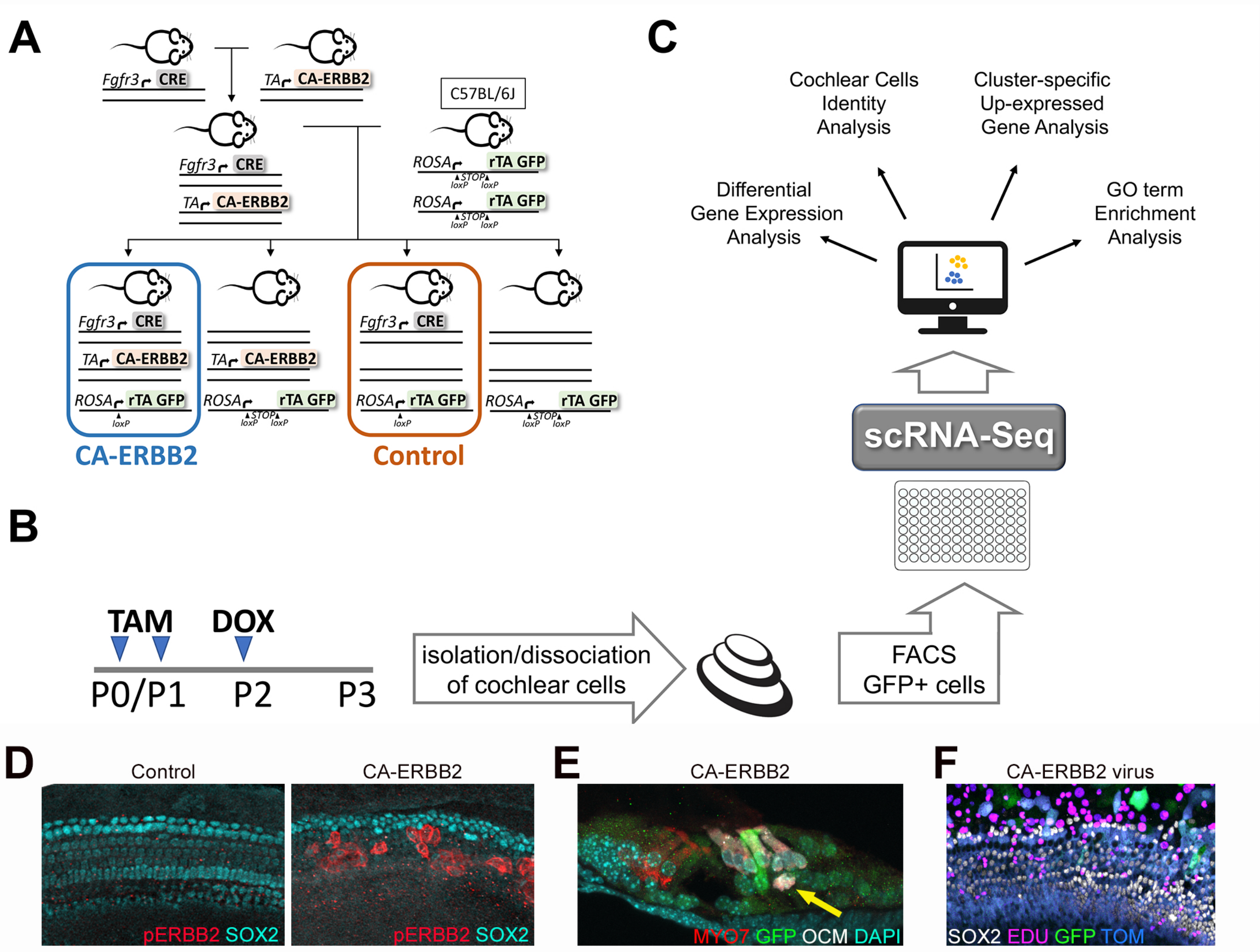

Supplementary Figure 1. Study design workflow for scRNA-seq analysis of transcriptomes of cochlear supporting cells expressing ERBB2.
(A) Breeding strategy to generate mice with CA-ERBB2 transgene inducible in cochlear supporting cells. (B) Timeline of tamoxifen (TAM) and doxycycline (DOX) injections in neonatal mice, and major steps in processing samples for scRNA-seq analysis. (C) Computational data set analysis of Control and CA-ERBB2 cells. (D-F) Example images from the previously published dataset that illustrate the indirect effects of CA-ERBB2 expression. (D) p-ERBB2 (red) is not evident in control cochlea (left) in the SOX2+ supporting cell layer (cyan), whereas in CA-ERBB2 cochleae (right), scattered pERBB2+ cells (red) correlate with a loss of SOX2 expression one day after dox induction. (E) An oblique section through a CA-ERBB2 cochlea shows a row of outer hair cells expressing MYO7 (red) and OCM (white), with a single GFP+ (green, marker for CA-ERBB2) presumptive Deiter cell. A nearby cell with its nucleus in the supporting cell layer co-expresses MYO7 and OCM (arrow), 14 days after dox induction. (F) A cochlea derived from a Sox2-iCRE/ROSA-flox-Tomato was cultured with hydroxtamoxifen to induce Tomato (blue) in supporting cells and then infected with Ad5-GFP-CA-ERBB2 (green). Dividing supporting cells take up EdU (magenta) and have down-regulated SOX2 protein (white). All methods and quantification for these examples can be found in (Zhang et al, 2019).

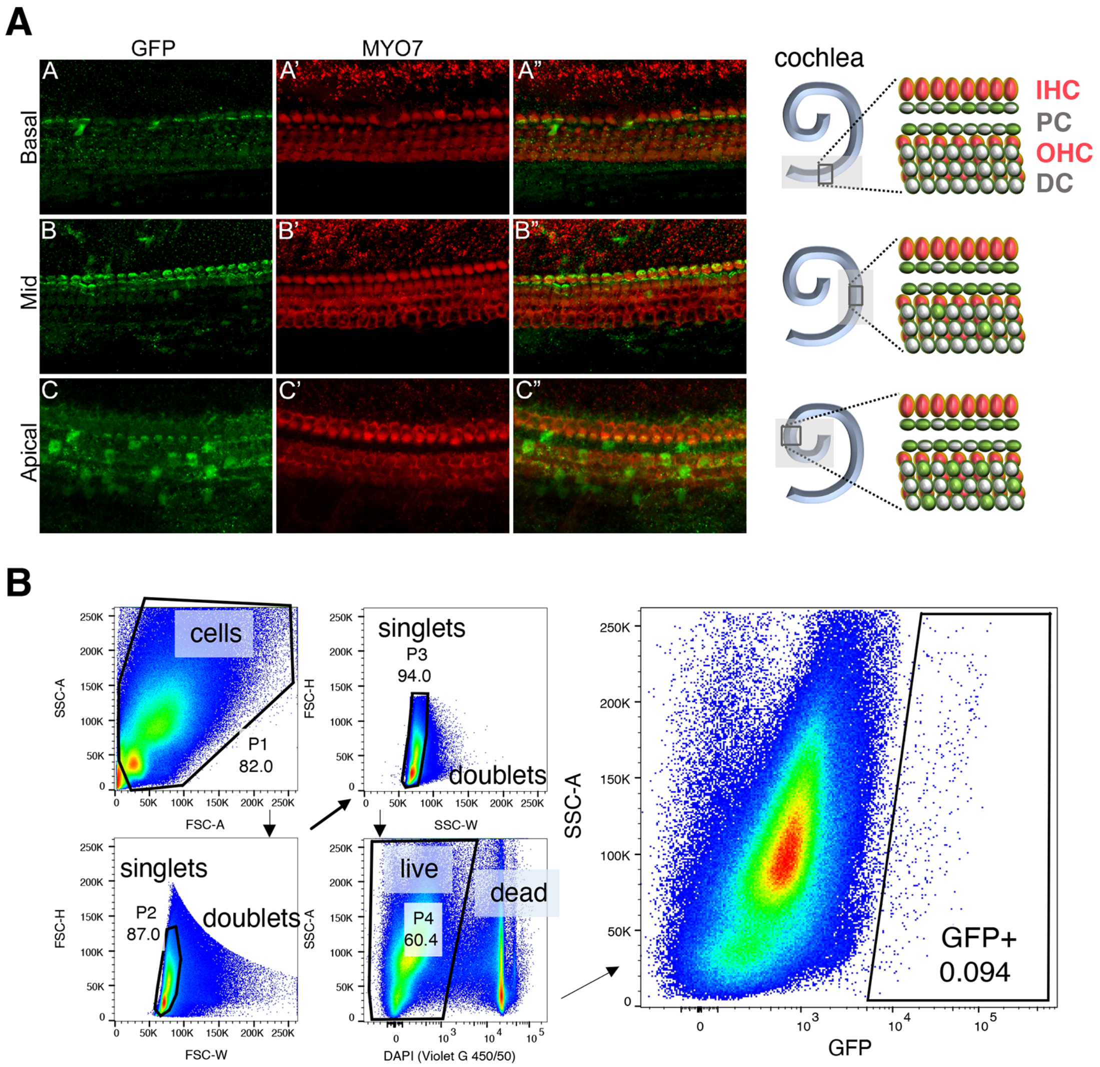

Figure S2. GFP labeling of supporting cells in the organ of Corti for FACS analysis. The iCre gene under control of Fgfr3 promoter is mostly expressed in supporting cells, such as Deiter cells, Pillar cells and Hensen cells. Two injections of tamoxifen at P0 and P1 allow activation of iCRE and activation of GFP expression mostly in apical region of the organ of Corti. (A) Shown are confocal images of a whole mount preparations of the organ of Corti from apical, mid and basal portions. Samples were collected at P3. Both, IHC and OHC are labelled with antibodies to MYO-VIIA (MYO7). (B) Shown are flow cytometry plots with gating of cochlear cells dissociated from P3 pups for FACS purification of GFP+ cells. P1, initial gating of cells to exclude debris. P2 and P3, gating based on forward scatter height (FSC-H), forward scatter width (FSC-W) and side scatter width (SSC-W) for doublets discrimination to select single cells. P4, gating of live cells based on negative staining with DAPI. GFP+ population is selected for sorting from the DAPI-negative cells (P4).

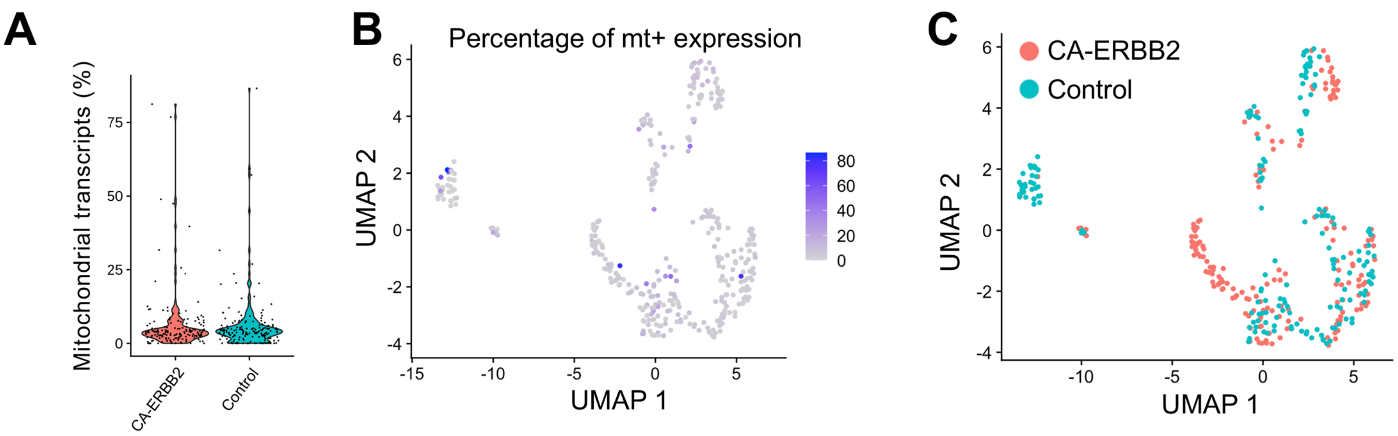

Supplementary Figure 3. Additional quality control metrics of scRNA-seq dataset. (A) Violin plots showing similar distributions observed for cells from Control and CA-ERBB2 samples in percentage of reads from mitochondrial transcripts per cell. Most of the cells are identified as intact cells based on a low percentage of reads from mitochondrial transcripts, as anticipated by their exclusion of a cell impermeant dye. (B) UMAP plots showing distribution of percentage of mitochondrial gene (mt) expression in cells. (C) UMAP plot of CA-ERBB2 and Control cells in clusters for comparison.

Supplementary Figure S4. UMAP plots and violin plots for marker gene transcripts identifying cochlear cells among ten clusters. Expression of gene markers was analyzed for Deiter cells, rows 1 and 2 (**A**), row 3 (**B**), Hensen cells (**C**), Inner Pillar cells (**D**), Outer Pillar cells (**E**), Inner Hair cells (**F**), Outer Hair cells (**G**), Lateral Greater Epithelial Ridge cells, group 1 (**H**), group 2 (**I**), group 3 (**J**), Medial Greater Epithelial Ridge cells (**K**), Inner Sulcus cells (**L**), Outer Sulcus cells (**M**), Interdental cells (**N**), and cells expressing Oc90 (**O**). Statistical analyses were performed with R version 4.0.5.

A

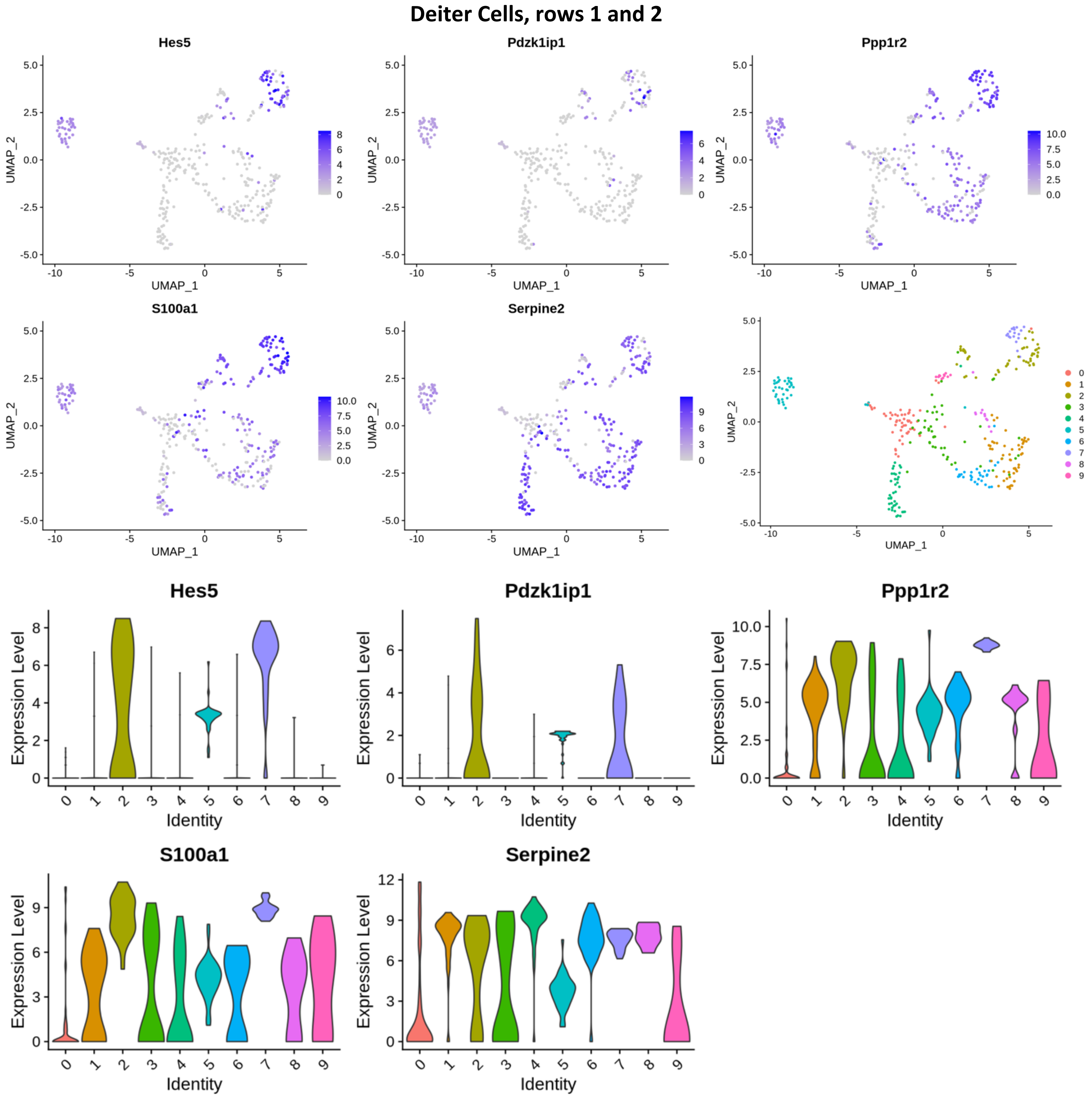

B

Deiter Cells, row 3

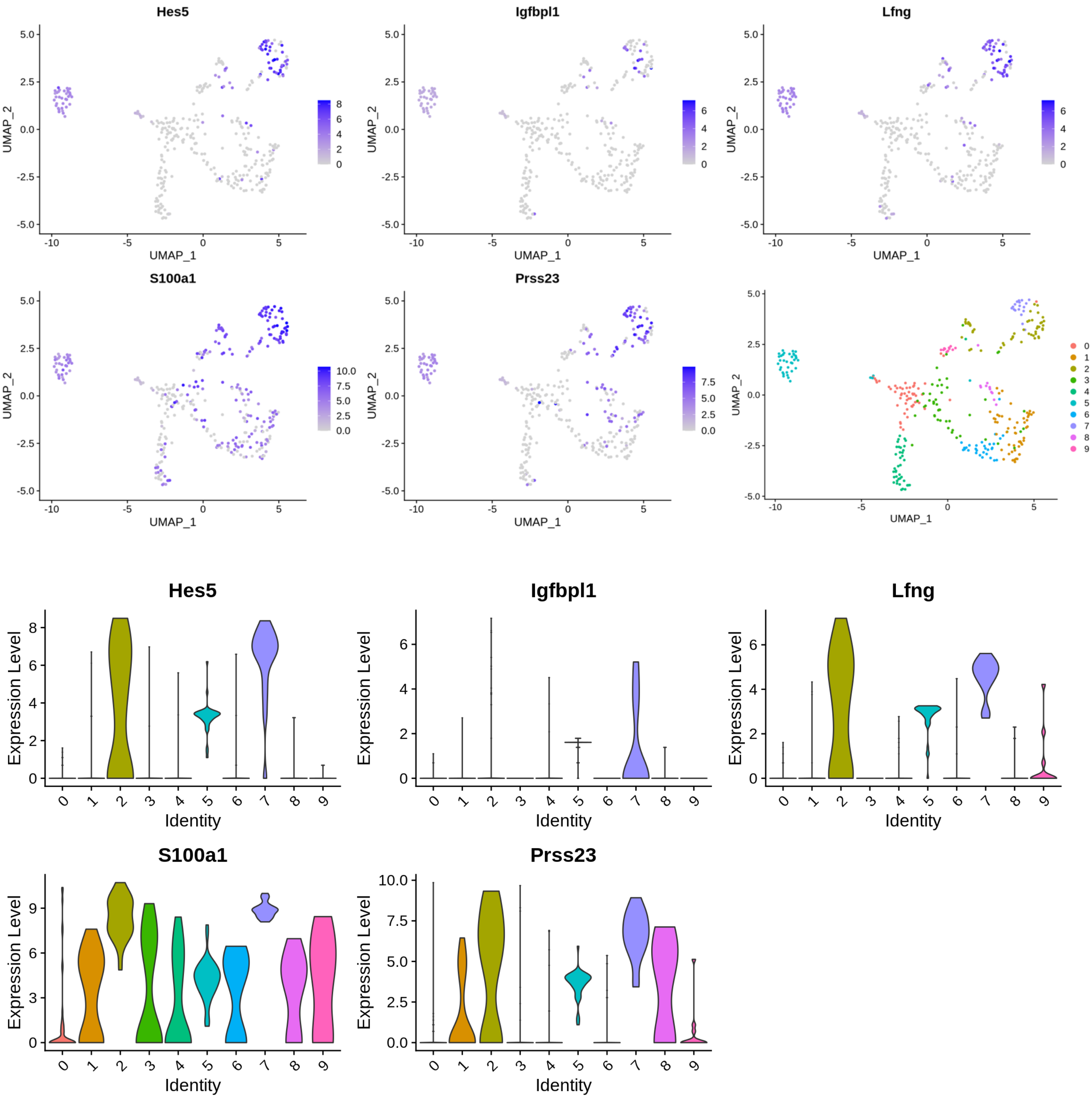

C

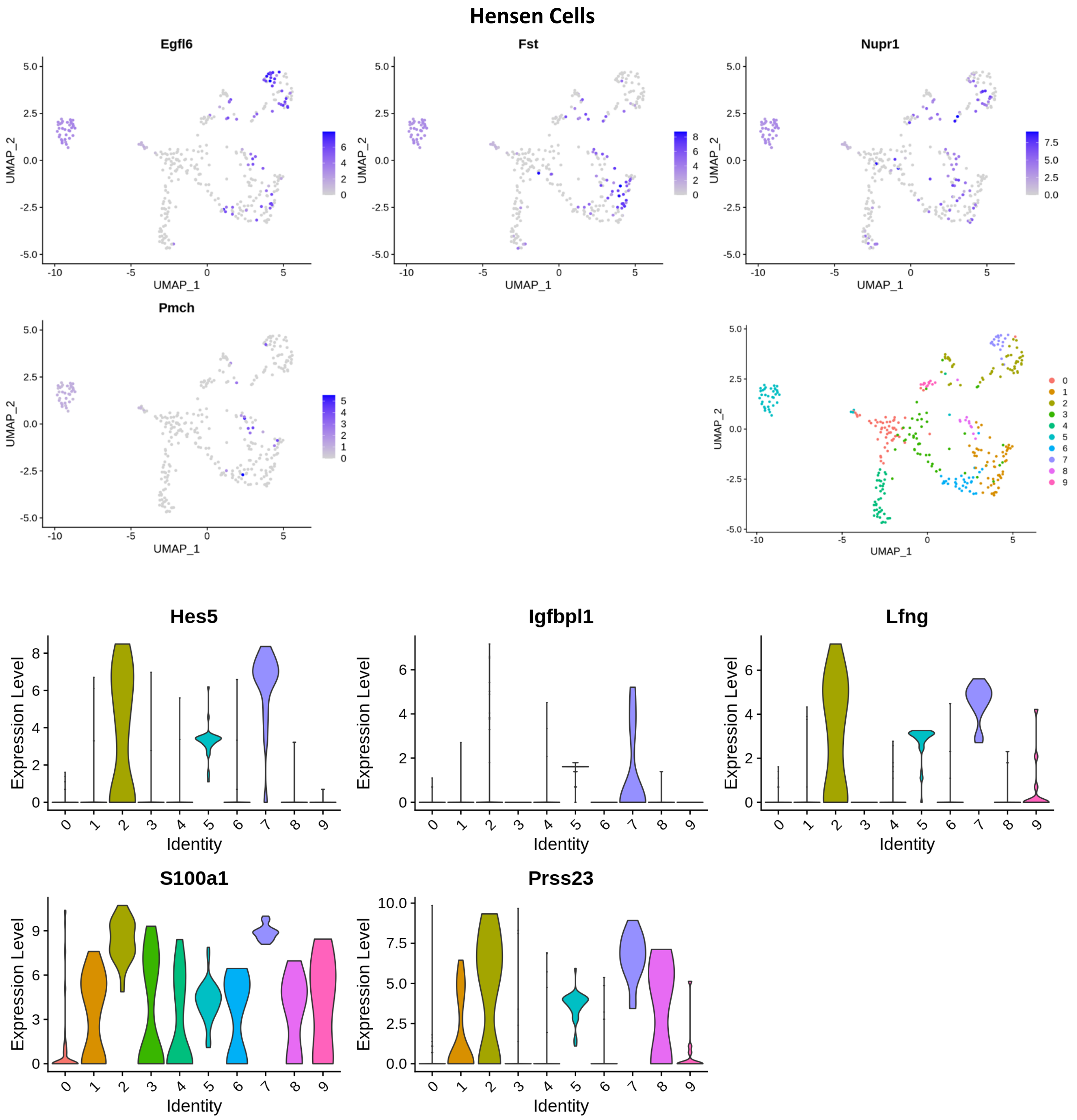
Hensen Cells
Egfl6
Fst
Nupr1
Pmch
UMAP_1
UMAP_2
Hes5
Igfbpl1
Lfng
S100a1
Prss23
Expression Level
Identity

D

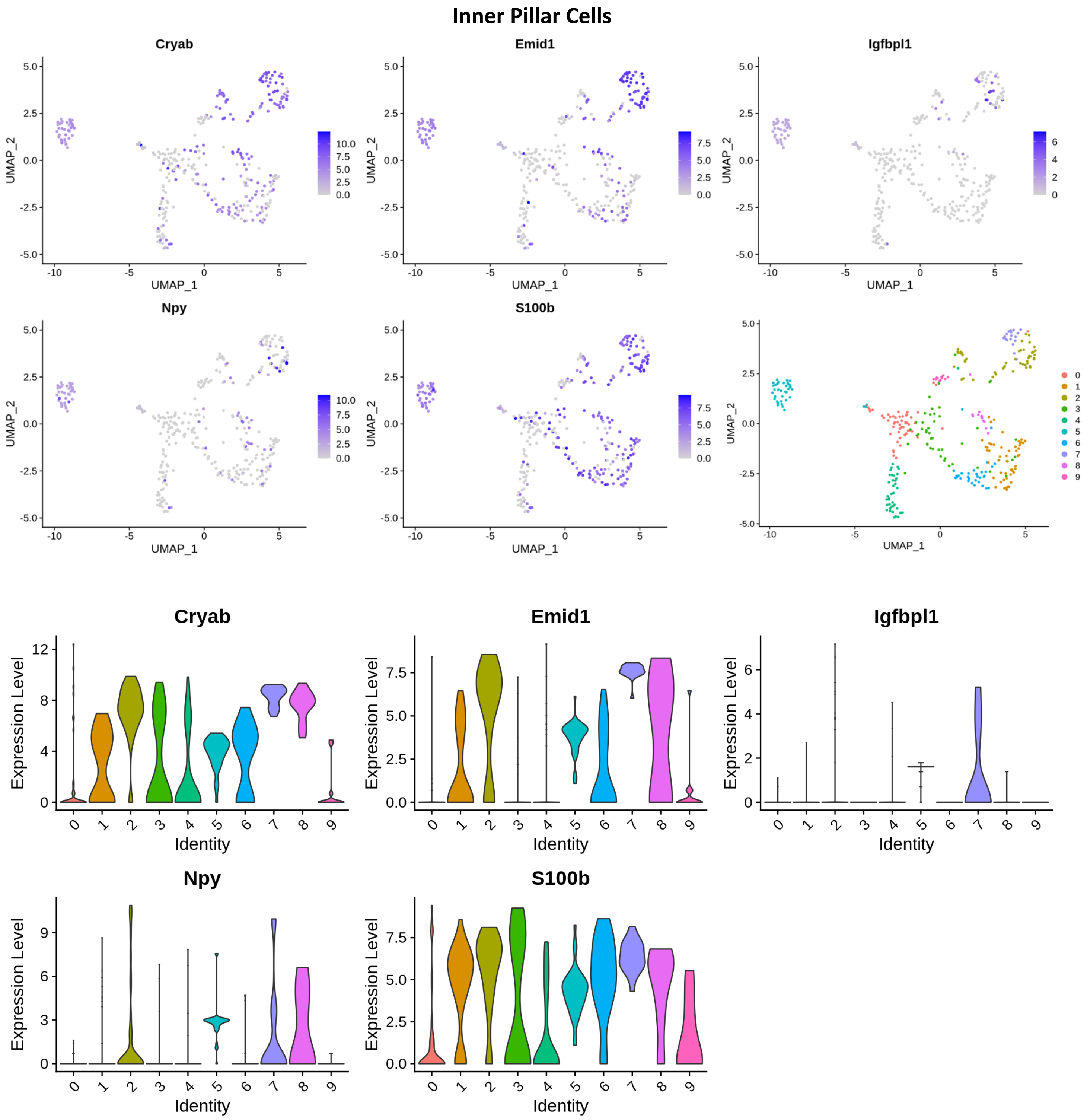

Inner Pillar Cells
Cryab
Emid1
Igfbpl1
Npy
S100b
UMAP_1
UMAP_2
Expression Level
Identity

E

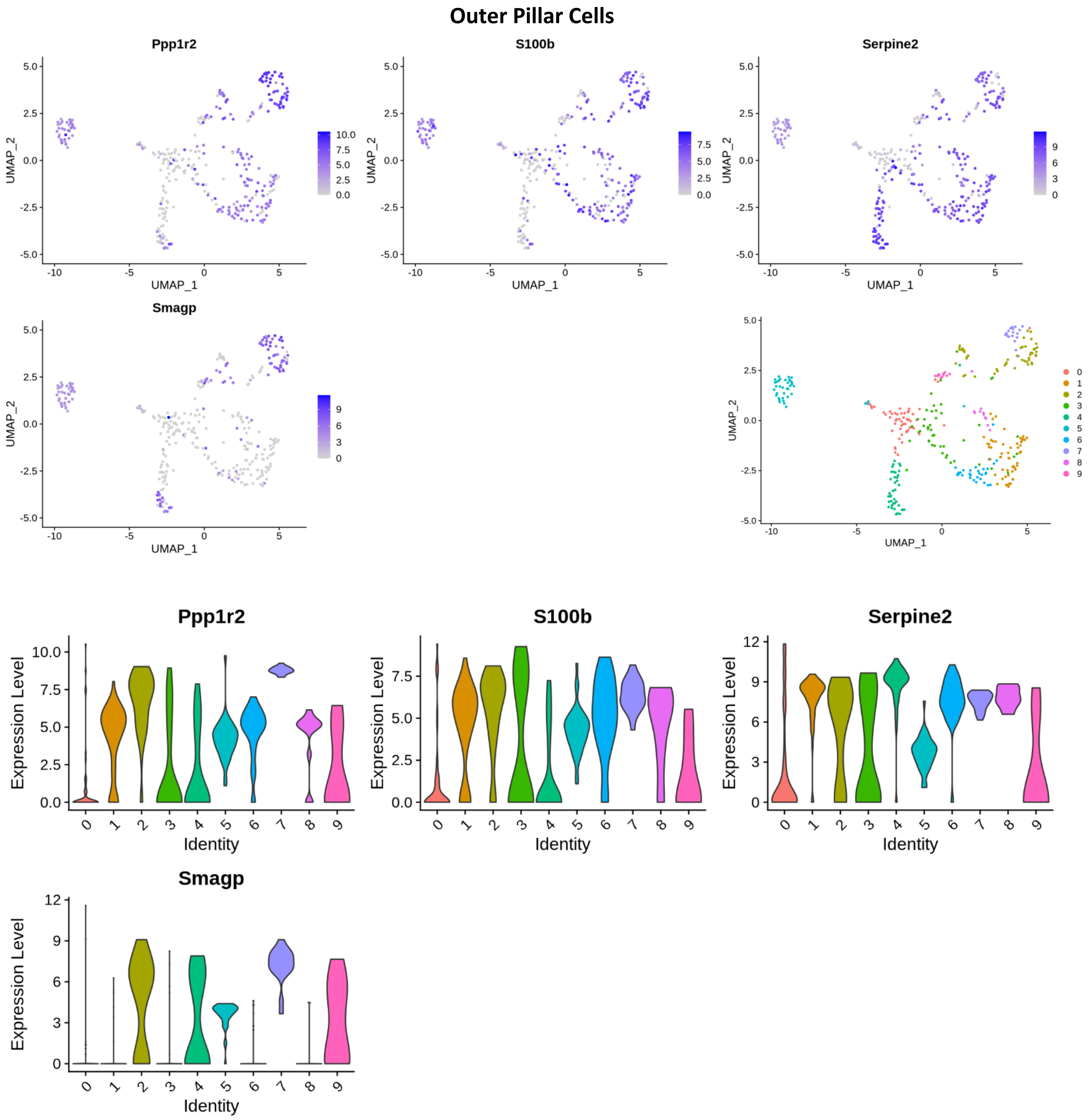

Outer Pillar Cells
Ppp1r2
S100b
Serpine2
Smagp
UMAP_1
UMAP_2
Expression Level
Identity
0
1
2
3
4
5
6
7
8
9

F

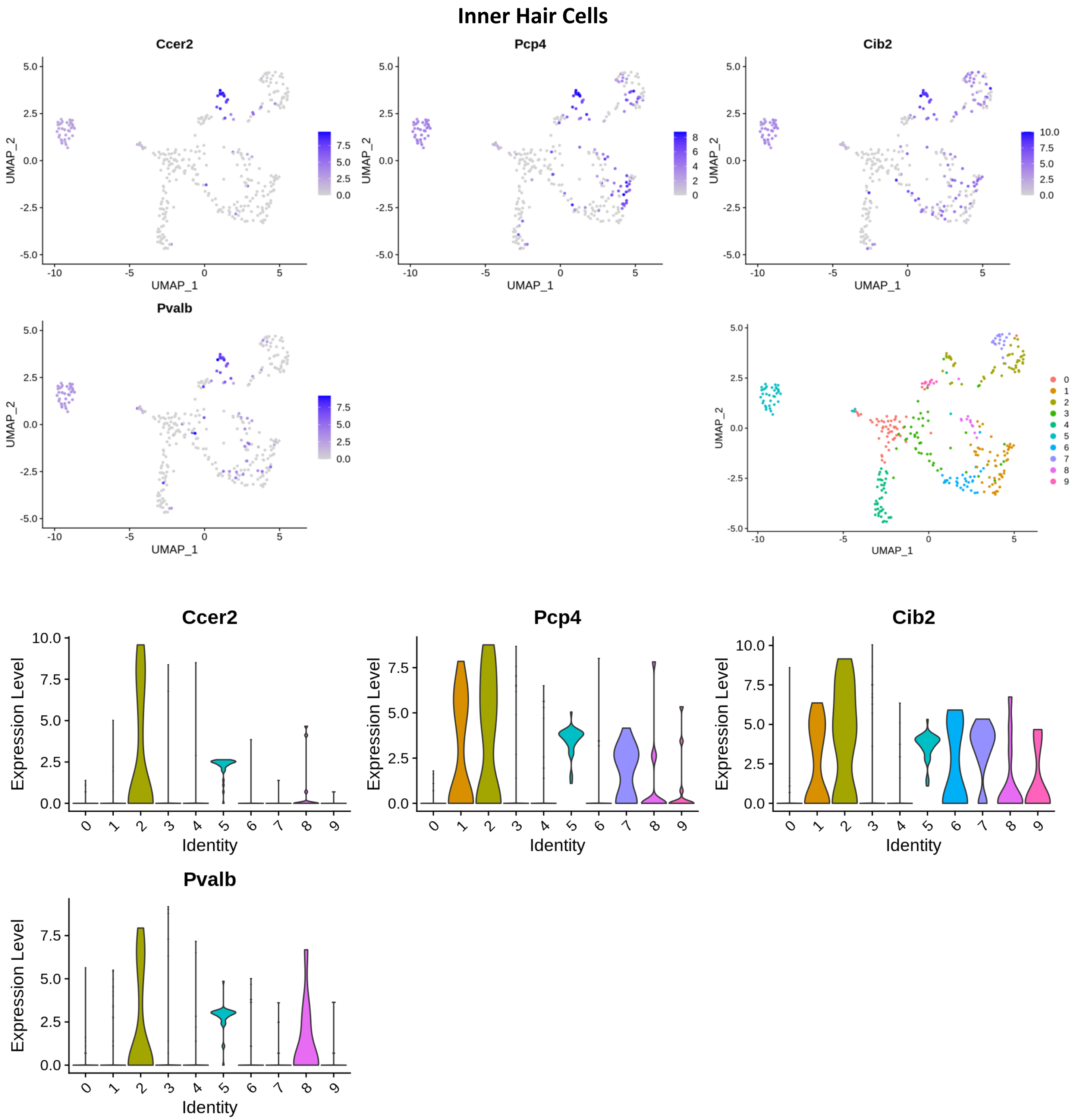
Inner Hair Cells
Ccer2
Pcp4
Cib2
Pvalb
UMAP_1
UMAP_2
Expression Level
Identity

G

Outer Hair Cells

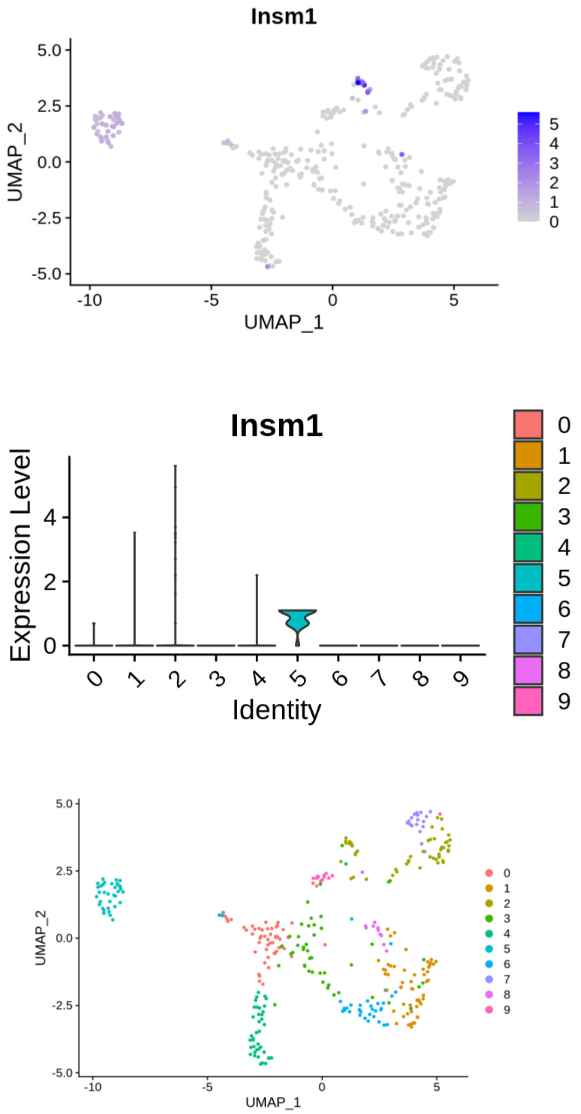

H

Lateral Greater Epithelial Ridge Cells, group 1

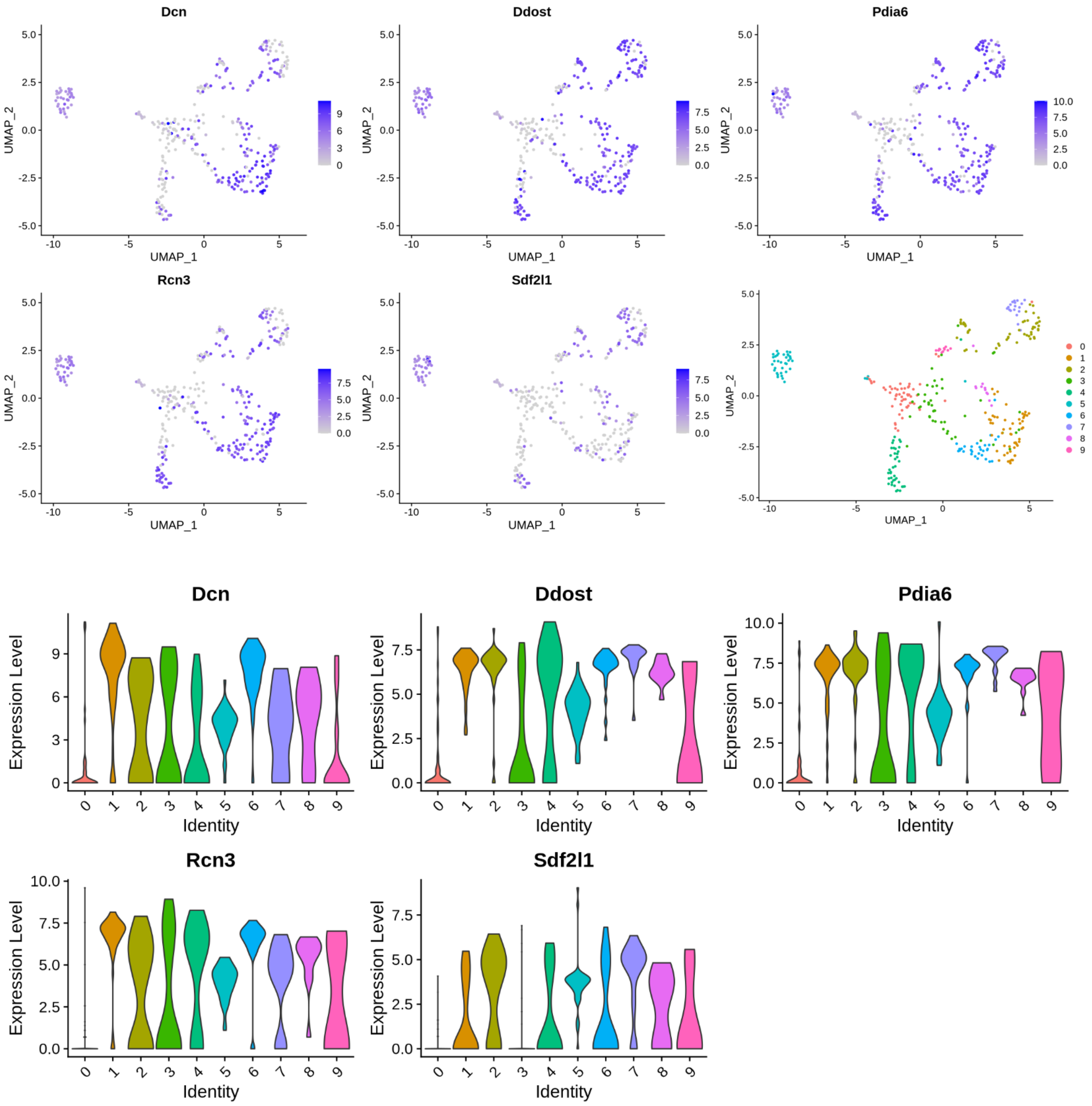

I

Lateral Greater Epithelial Ridge Cells, group 2

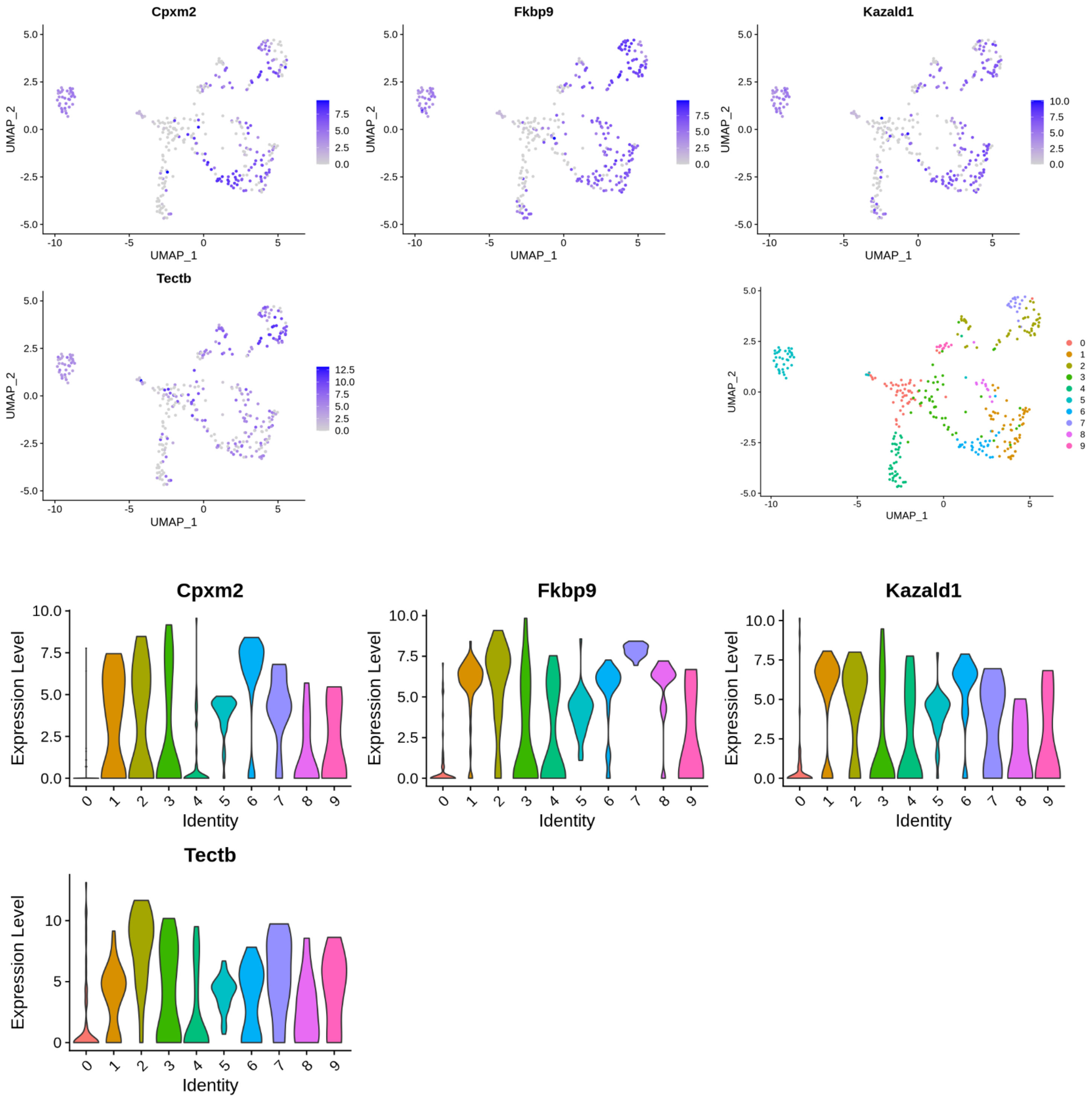

J

Lateral Greater Epithelial Ridge Cells, group 3

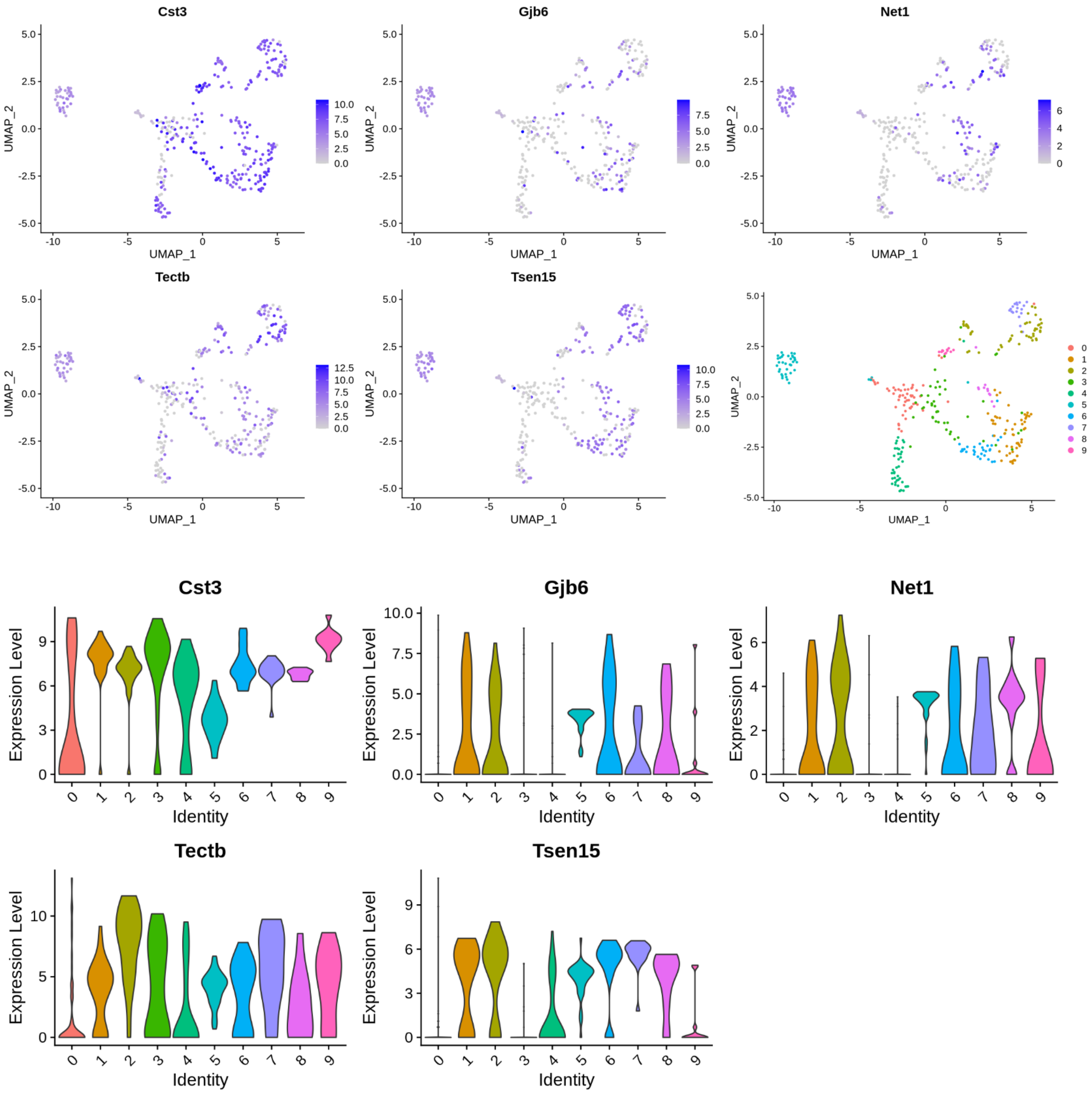

K

Medial Greater Epithelial Ridge Cells

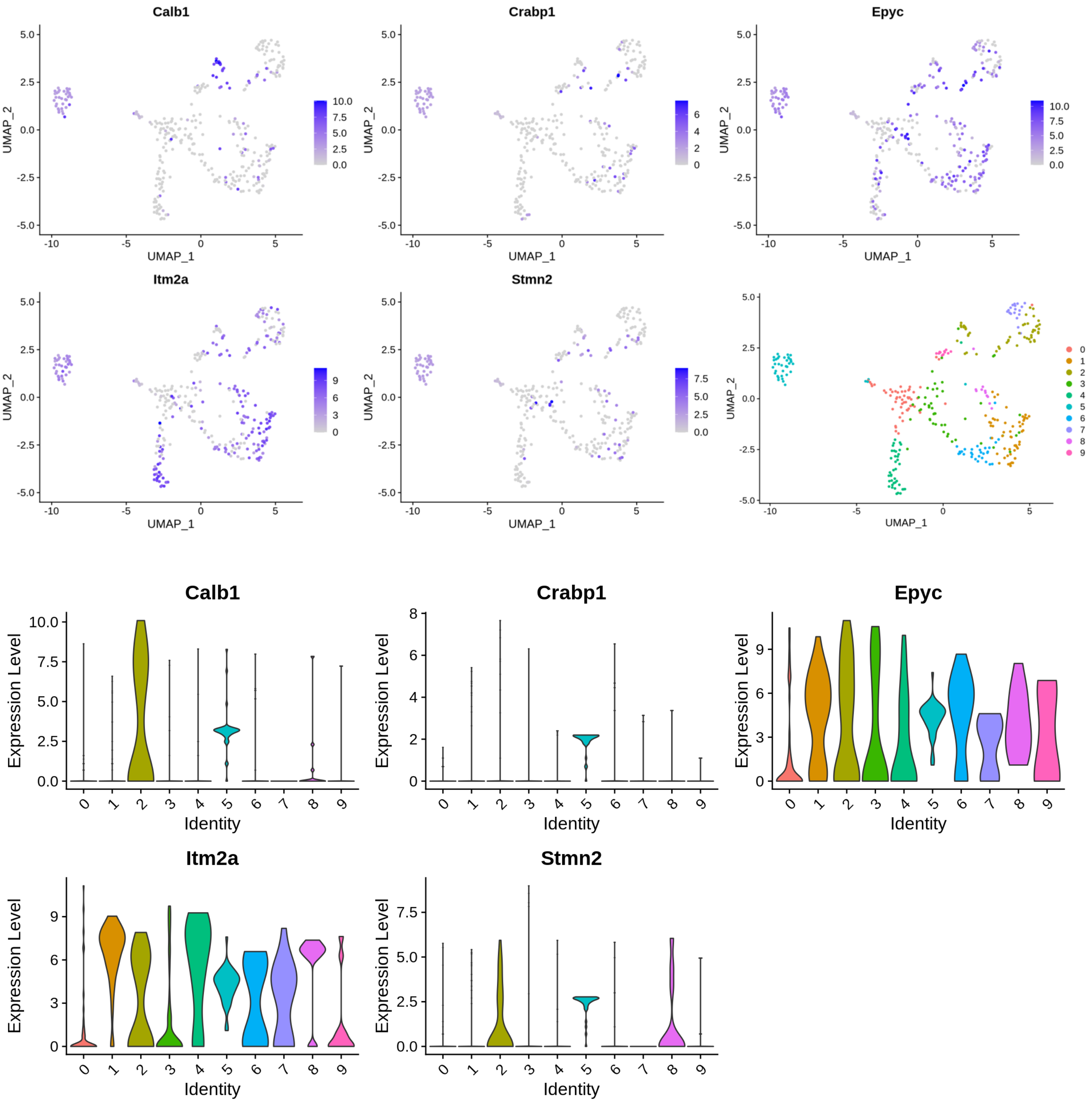

L

Inner Sulcus Cells

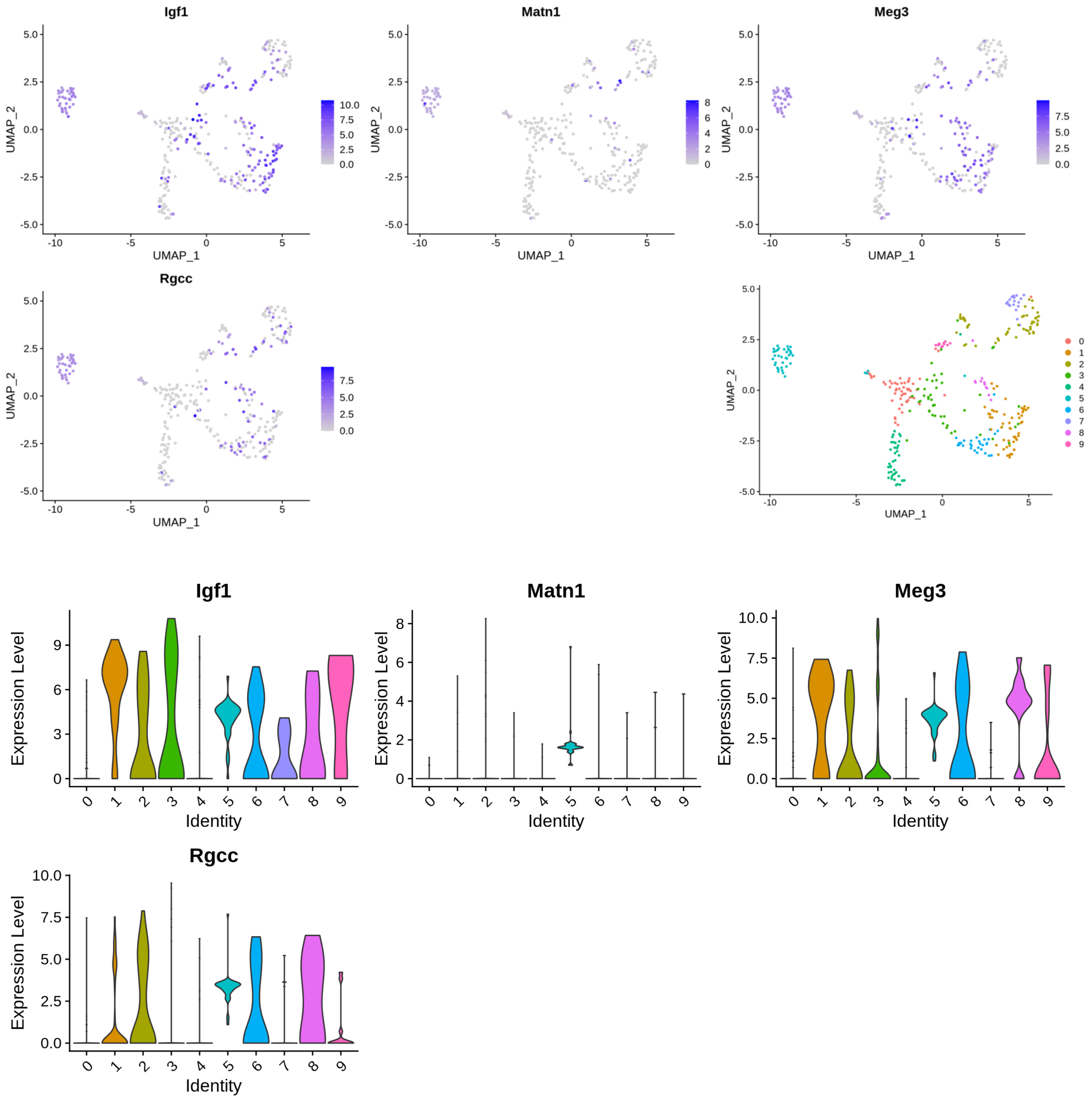

M

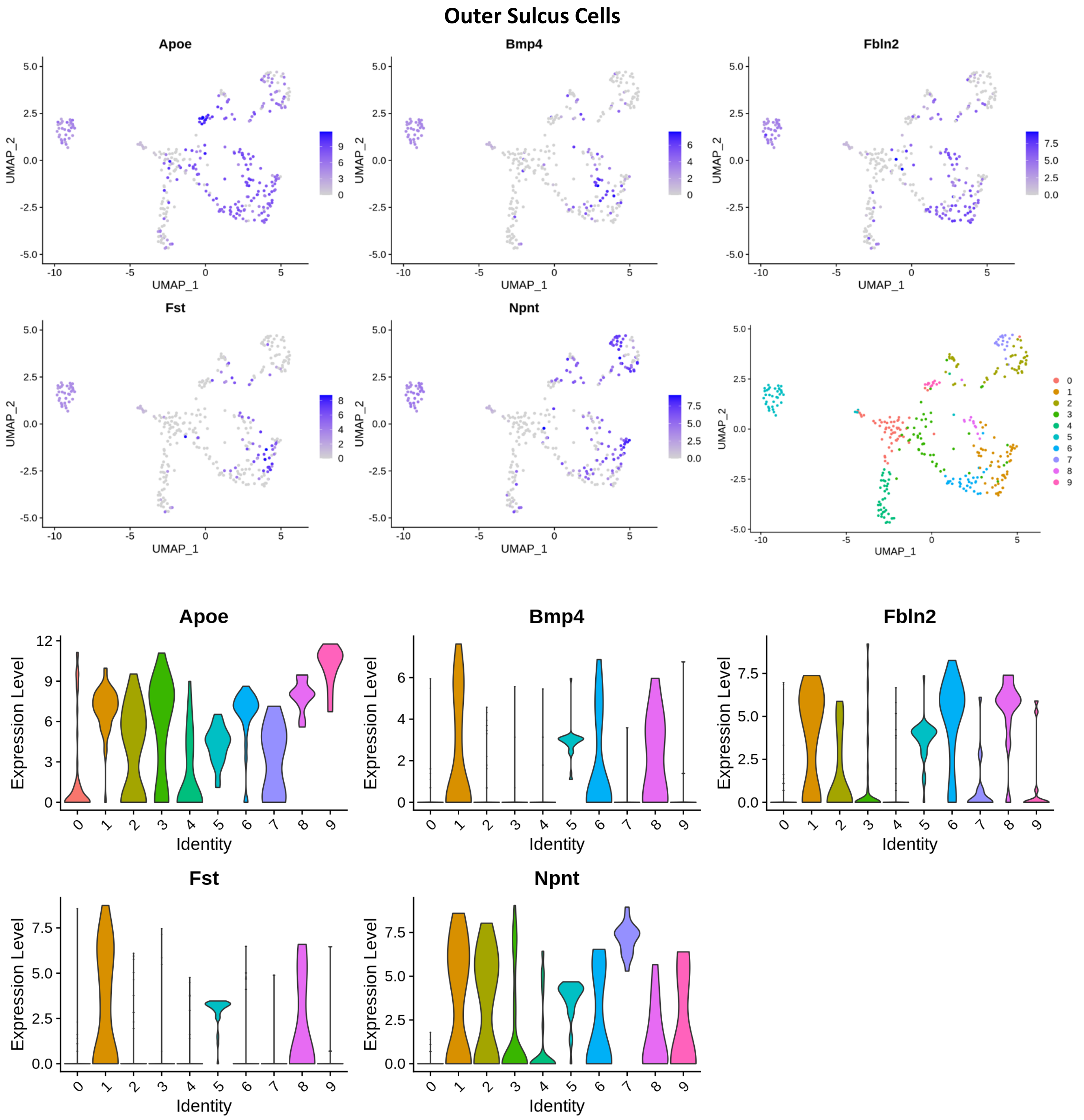

Outer Sulcus Cells
Apoe
Bmp4
Fbln2
Fst
Npnt
UMAP_1
UMAP_2
Expression Level
Identity
0
1
2
3
4
5
6
7
8
9

N

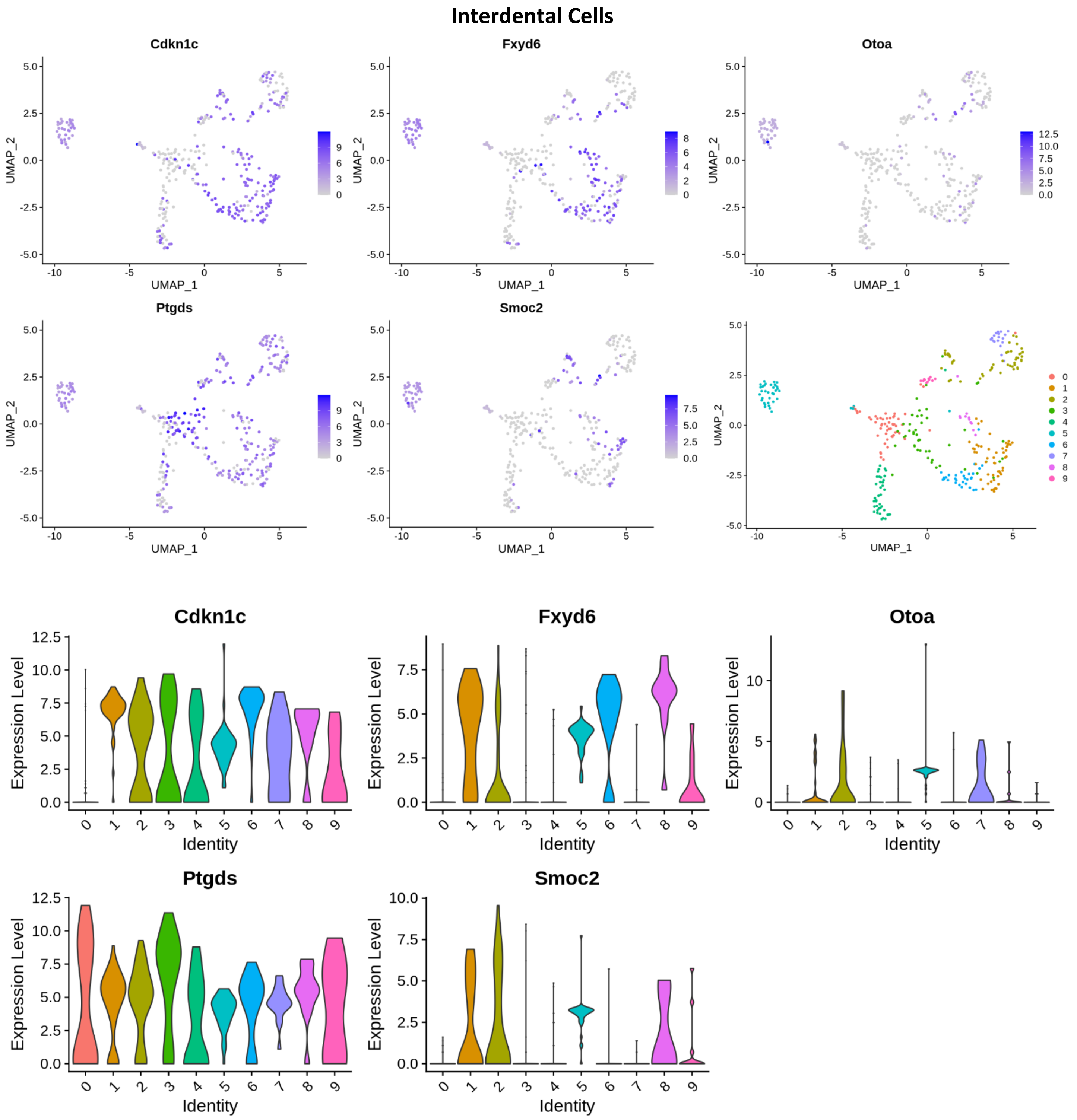
Interdental Cells
Cdkn1c
Fxyd6
Otoa
Ptgds
Smoc2
UMAP_1
UMAP_2
Expression Level
Identity

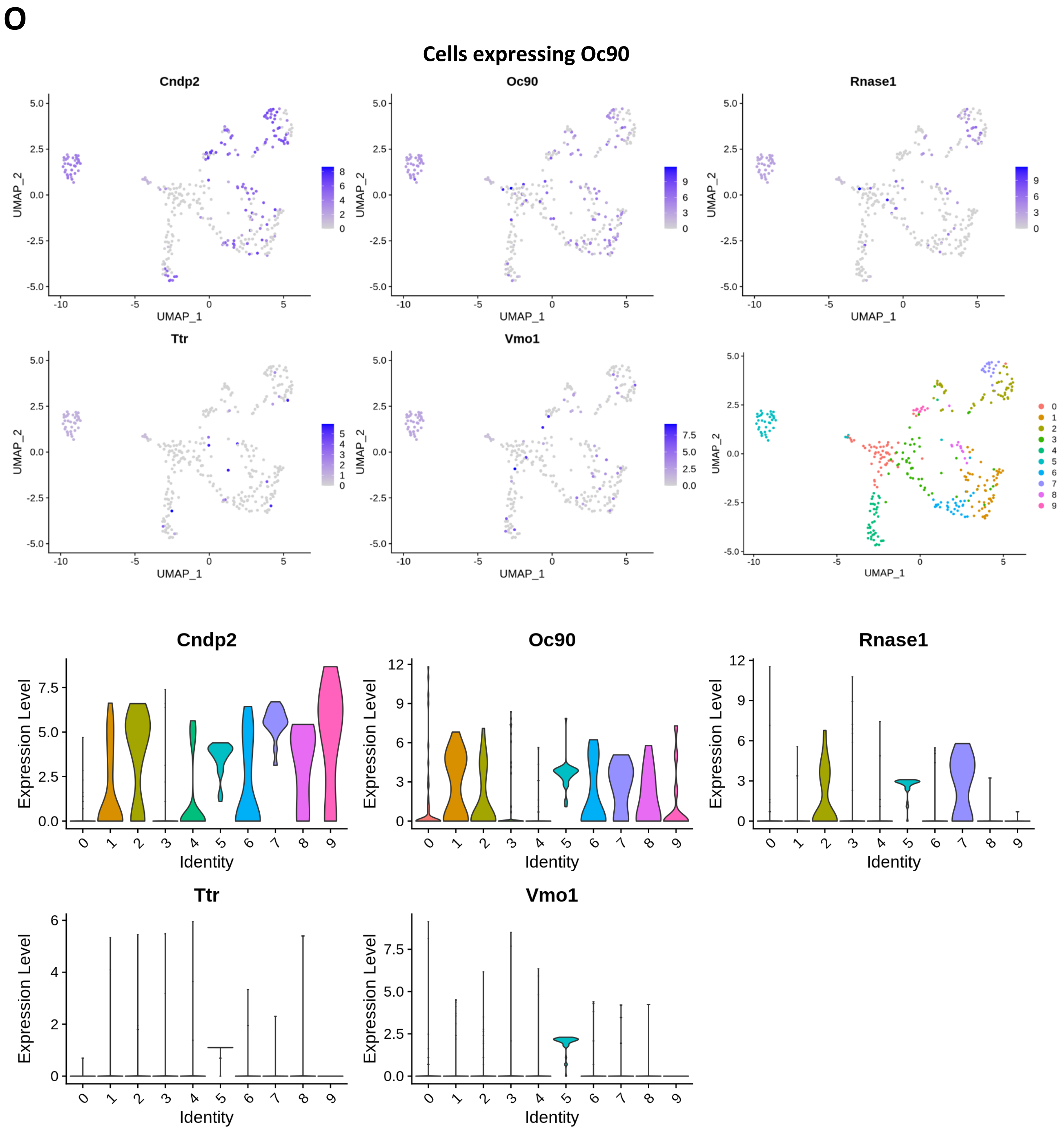
O
Cells expressing Oc90
Cndp2
Oc90
Rnase1
Ttr
Vmo1
UMAP_1
UMAP_2
Expression Level
Identity
0
1
2
3
4
5
6
7
8
9

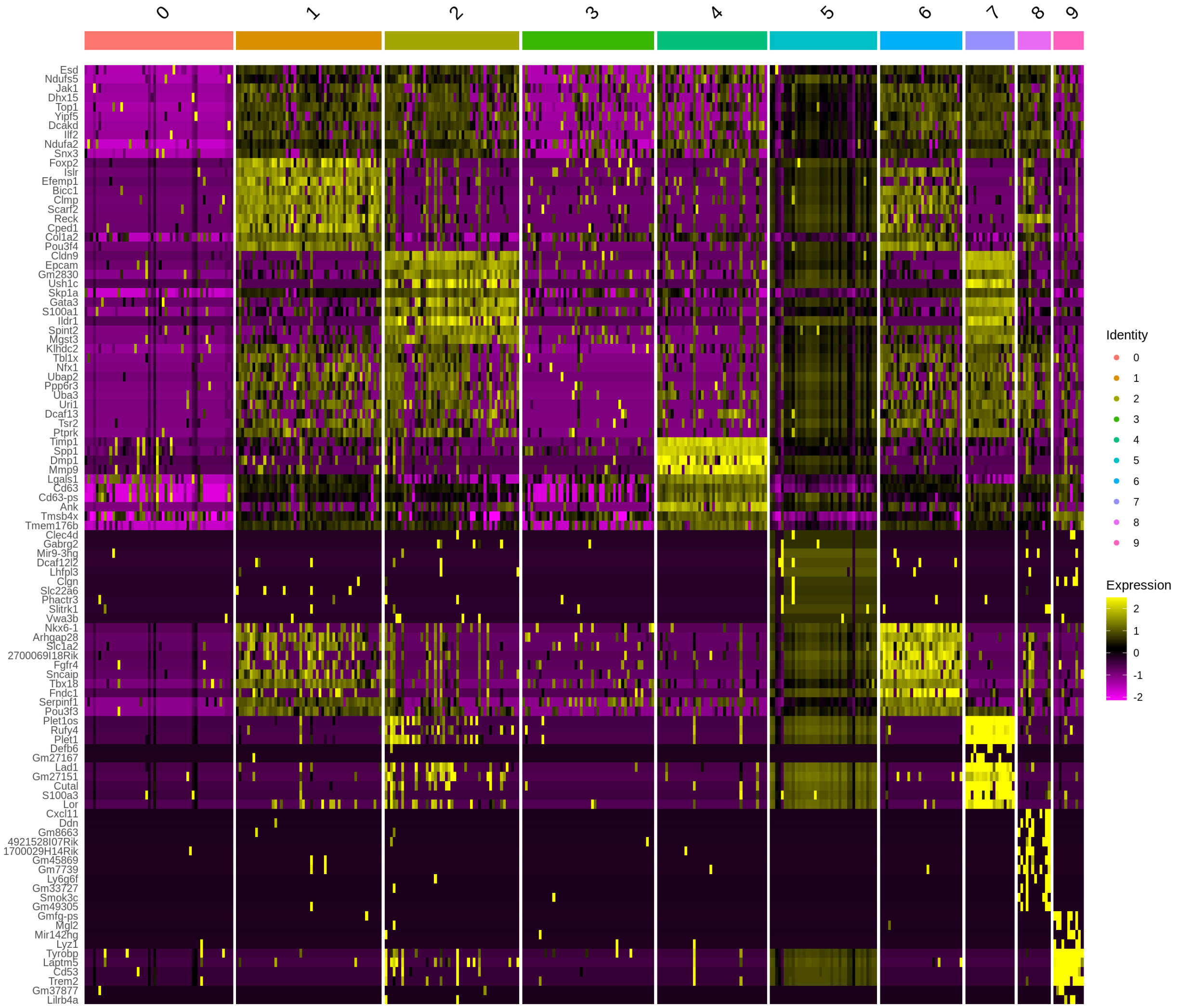

Supplementary Figure 5. Heatmap depicting the top 10 genes expressed by 10 different clusters of cochlear SCs.

Clusters S4 and S7 are composed of CA-ERBB2 cells only. Cells from each cluster are arrayed along the horizontal axis and genes are arrayed along vertical axis. Clusters are distinguished by the color bars.

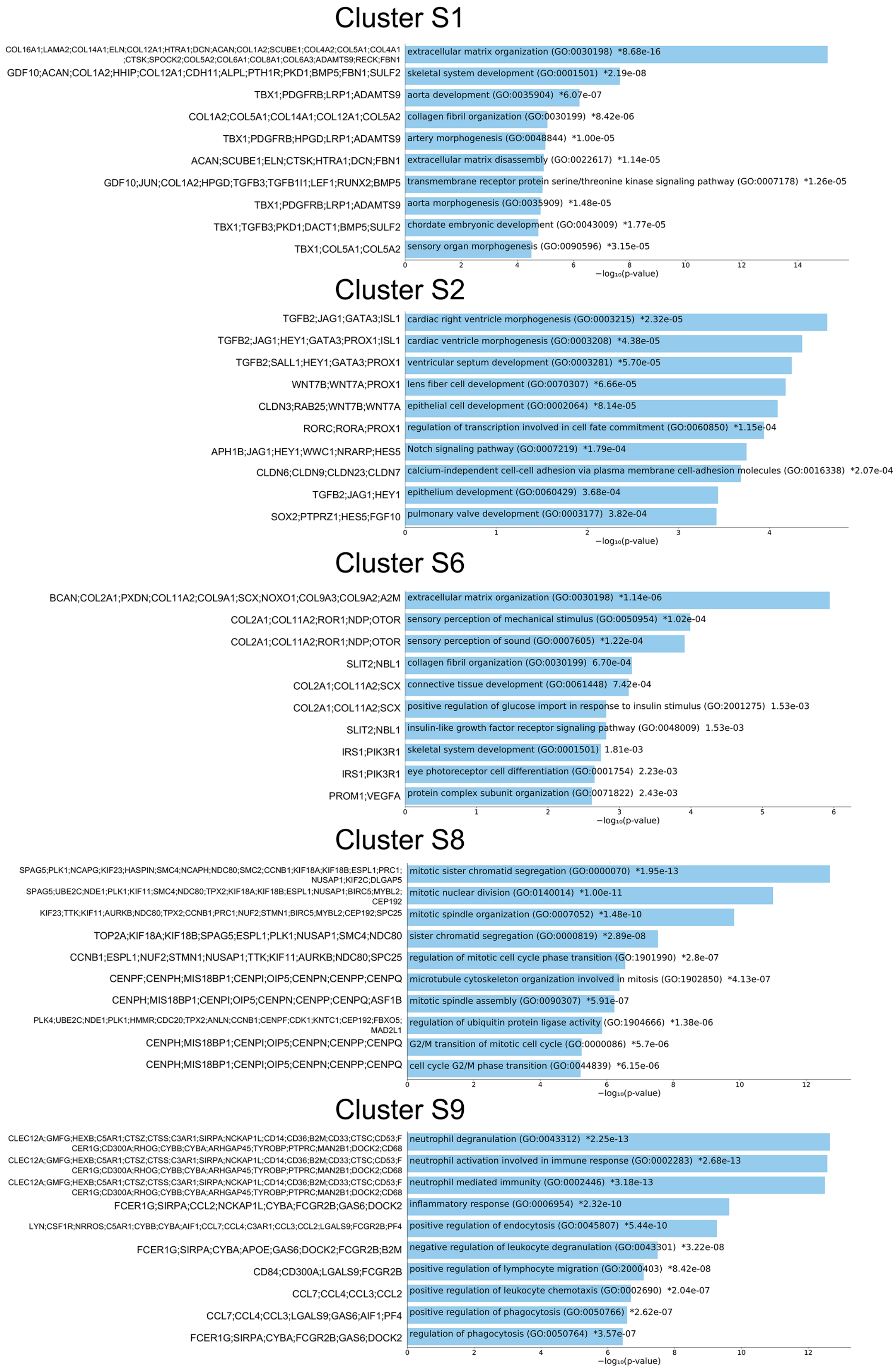
Cluster S1
COL16A1;LAMA2;COL14A1;ELN;COL12A1;HTRA1;DCN;ACAN;COL1A2;SCUBE1;COL4A2;COL5A1;COL4A1;CTSK;SPOCK2;COL5A2;COL6A1;COL8A1;COL6A3;ADAMTS9;RECK;FBN1
extracellular matrix organization (GO:0030198) *8.68e-16
GDF10;ACAN;COL1A2;HHIP;COL12A1;CDH11;ALPL;PTH1R;PKD1;BMP5;FBN1;SULF2
skeletal system development (GO:0001501) *2.19e-08
TBX1;PDGFRB;LRP1;ADAMTS9
aorta development (GO:0035904) *6.07e-07
COL1A2;COL5A1;COL14A1;COL12A1;COL5A2
collagen fibril organization (GO:0030199) *8.42e-06
TBX1;PDGFRB;HPGD;LRP1;ADAMTS9
artery morphogenesis (GO:0048844) *1.00e-05
ACAN;SCUBE1;ELN;CTSK;HTRA1;DCN;FBN1
extracellular matrix disassembly (GO:0022617) *1.14e-05
GDF10;JUN;COL1A2;HPGD;TGFB3;TGFB1I1;LEF1;RUNX2;BMP5
transmembrane receptor protein serine/threonine kinase signaling pathway (GO:0007178) *1.26e-05
TBX1;PDGFRB;LRP1;ADAMTS9
aorta morphogenesis (GO:0035909) *1.48e-05
TBX1;TGFB3;PKD1;DACT1;BMP5;SULF2
chordate embryonic development (GO:0043009) *1.77e-05
TBX1;COL5A1;COL5A2
sensory organ morphogenesis (GO:0090596) *3.15e-05
0 2 4 6 8 10 12 14
−log₁₀(p-value)
Cluster S2
TGFB2;JAG1;GATA3;ISL1
cardiac right ventricle morphogenesis (GO:0003215) *2.32e-05
TGFB2;JAG1;HEY1;GATA3;PROX1;ISL1
cardiac ventricle morphogenesis (GO:0003208) *4.38e-05
TGFB2;SALL1;HEY1;GATA3;PROX1
ventricular septum development (GO:0003281) *5.70e-05
WNT7B;WNT7A;PROX1
lens fiber cell development (GO:0070307) *6.66e-05
CLDN3;RAB25;WNT7B;WNT7A
epithelial cell development (GO:0002064) *8.14e-05
RORC;RORA;PROX1
regulation of transcription involved in cell fate commitment (GO:0060850) *1.15e-04
APH1B;JAG1;HEY1;WWC1;NRARP;HES5
Notch signaling pathway (GO:0007219) *1.79e-04
CLDN6;CLDN9;CLDN23;CLDN7
calcium-independent cell-cell adhesion via plasma membrane cell-adhesion molecules (GO:0016338) *2.07e-04
TGFB2;JAG1;HEY1
epithelium development (GO:0060429) 3.68e-04
SOX2;PTPRZ1;HES5;FGF10
pulmonary valve development (GO:0003177) 3.82e-04
0 1 2 3 4
−log₁₀(p-value)
Cluster S6
BCAN;COL2A1;PXDN;COL11A2;COL9A1;SCX;NOXO1;COL9A3;COL9A2;A2M
extracellular matrix organization (GO:0030198) *1.14e-06
COL2A1;COL11A2;ROR1;NDP;OTOR
sensory perception of mechanical stimulus (GO:0050954) *1.02e-04
COL2A1;COL11A2;ROR1;NDP;OTOR
sensory perception of sound (GO:0007605) *1.22e-04
SLIT2;NBL1
collagen fibril organization (GO:0030199) 6.70e-04
COL2A1;COL11A2;SCX
connective tissue development (GO:0061448) 7.42e-04
COL2A1;COL11A2;SCX
positive regulation of glucose import in response to insulin stimulus (GO:2001275) 1.53e-03
SLIT2;NBL1
insulin-like growth factor receptor signaling pathway (GO:0048009) 1.53e-03
IRS1;PIK3R1
skeletal system development (GO:0001501) 1.81e-03
IRS1;PIK3R1
eye photoreceptor cell differentiation (GO:0001754) 2.23e-03
PROM1;VEGFA
protein complex subunit organization (GO:0071822) 2.43e-03
0 1 2 3 4 5 6
−log₁₀(p-value)
Cluster S8
SPAG5;PLK1;NCAPG;KIF23;HASPIN;SMC4;NCAPH;NDC80;SMC2;CCNB1;KIF18A;KIF18B;ESPL1;PRC1;NUSAP1;KIF2C;DLGAP5
mitotic sister chromatid segregation (GO:0000070) *1.95e-13
SPAG5;UBE2C;NDE1;PLK1;KIF11;SMC4;NDC80;TPX2;KIF18A;KIF18B;ESPL1;NUSAP1;BIRC5;MYBL2;CEP192
mitotic nuclear division (GO:0140014) *1.00e-11
KIF23;TTK;KIF11;AURKB;NDC80;TPX2;CCNB1;PRC1;NUF2;STMN1;BIRC5;MYBL2;CEP192;SPC25
mitotic spindle organization (GO:0007052) *1.48e-10
TOP2A;KIF18A;KIF18B;SPAG5;ESPL1;PLK1;NUSAP1;SMC4;NDC80
sister chromatid segregation (GO:0000819) *2.89e-08
CCNB1;ESPL1;NUF2;STMN1;NUSAP1;TTK;KIF11;AURKB;NDC80;SPC25
regulation of mitotic cell cycle phase transition (GO:1901990) *2.8e-07
CENPF;CENPH;MIS18BP1;CENPI;OIP5;CENPN;CENPP;CENPQ
microtubule cytoskeleton organization involved in mitosis (GO:1902850) *4.13e-07
CENPH;MIS18BP1;CENPI;OIP5;CENPN;CENPP;CENPQ;ASF1B
mitotic spindle assembly (GO:0090307) *5.91e-07
PLK4;UBE2C;NDE1;PLK1;HMMR;CDC20;TPX2;ANLN;CCNB1;CENPF;CDK1;KNTC1;CEP192;FBXO5;MAD2L1
regulation of ubiquitin protein ligase activity (GO:1904666) *1.38e-06
CENPH;MIS18BP1;CENPI;OIP5;CENPN;CENPP;CENPQ
G2/M transition of mitotic cell cycle (GO:0000086) *5.7e-06
CENPH;MIS18BP1;CENPI;OIP5;CENPN;CENPP;CENPQ
cell cycle G2/M phase transition (GO:0044839) *6.15e-06
0 2 4 6 8 10 12
−log₁₀(p-value)
Cluster S9
CLEC12A;GMFG;HEXB;C5AR1;CTSZ;CTSS;C3AR1;SIRPA;NCKAP1L;CD14;CD36;B2M;CD33;CTSC;CD53;FCER1G;CD300A;RHOG;CYBB;CYBA;ARHGAP45;TYROBP;PTPRC;MAN2B1;DOCK2;CD68
neutrophil degranulation (GO:0043312) *2.25e-13
CLEC12A;GMFG;HEXB;C5AR1;CTSZ;CTSS;C3AR1;SIRPA;NCKAP1L;CD14;CD36;B2M;CD33;CTSC;CD53;FCER1G;CD300A;RHOG;CYBB;CYBA;ARHGAP45;TYROBP;PTPRC;MAN2B1;DOCK2;CD68
neutrophil activation involved in immune response (GO:0002283) *2.68e-13
CLEC12A;GMFG;HEXB;C5AR1;CTSZ;CTSS;C3AR1;SIRPA;NCKAP1L;CD14;CD36;B2M;CD33;CTSC;CD53;FCER1G;CD300A;RHOG;CYBB;CYBA;ARHGAP45;TYROBP;PTPRC;MAN2B1;DOCK2;CD68
neutrophil mediated immunity (GO:0002446) *3.18e-13
FCER1G;SIRPA;CCL2;NCKAP1L;CYBA;FCGR2B;GAS6;DOCK2
inflammatory response (GO:0006954) *2.32e-10
LYN;CSF1R;NRROS;C5AR1;CYBB;CYBA;AIF1;CCL7;CCL4;C3AR1;CCL3;CCL2;LGALS9;FCGR2B;PF4
positive regulation of endocytosis (GO:0045807) *5.44e-10
FCER1G;SIRPA;CYBA;APOE;GAS6;DOCK2;FCGR2B;B2M
negative regulation of leukocyte degranulation (GO:0043301) *3.22e-08
CD84;CD300A;LGALS9;FCGR2B
positive regulation of lymphocyte migration (GO:2000403) *8.42e-08
CCL7;CCL4;CCL3;CCL2
positive regulation of leukocyte chemotaxis (GO:0002690) *2.04e-07
CCL7;CCL4;CCL3;LGALS9;GAS6;AIF1;PF4
positive regulation of phagocytosis (GO:0050766) *2.62e-07
FCER1G;SIRPA;CYBA;FCGR2B;GAS6;DOCK2
regulation of phagocytosis (GO:0050764) *3.57e-07
0 2 4 6 8 10 12
−log₁₀(p-value)

Figure S6. Top 10 significantly enriched Gene Ontology (GO) terms in different clusters. For each cluster, bar chart shows the top 10 enriched GO terms of biological process along with corresponding p-values (< 0.05). An asterisk (*) next to a p-value indicates the term also has a significant q-value (<0.05). The y-axis represents biological process, and the horizontal axis represents the number of genes, which are listed on the left side of the graph. Complete list of terms for each cluster is provided in Supplementary Data 4.

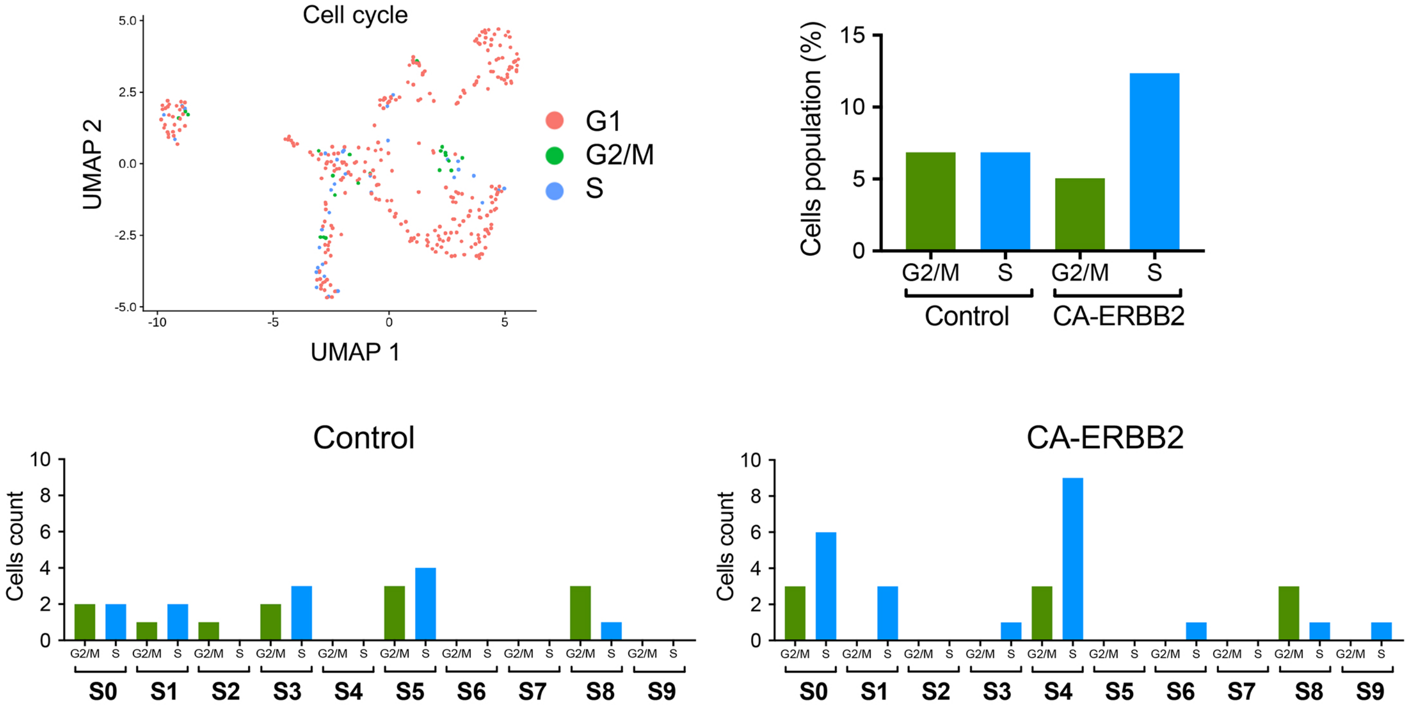

Figure S7. Cell cycle analysis. Top left, UMAP plot showing distribution of cells with markers identifying cell cycle phases G1, G2/M and S. Top right, bar chart showing proportion of Control cells and CA-ERBB2 cells in cell cycle phase G2/M and S. Bottom bar charts show distribution of Control cells and CA-ERBB2 cells in G2/M and S phase by cluster.

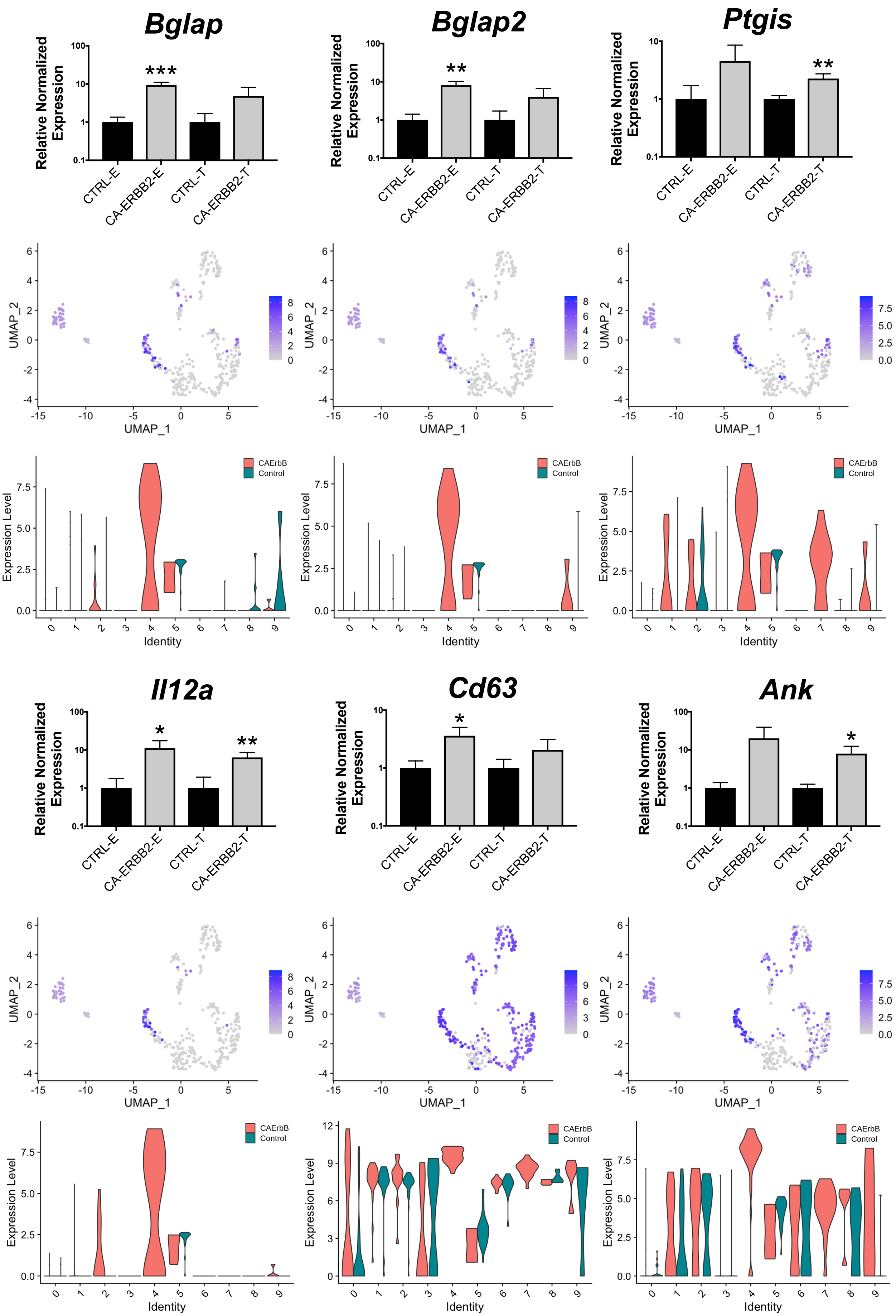

Bglap
Bglap2
Ptgis
Relative Normalized Expression
CTRL-E
CA-ERBB2-E
CTRL-T
CA-ERBB2-T
***
**
UMAP_1
UMAP_2
CAErbB
Control
Expression Level
Identity
Il12a
Cd63
Ank
*

Supplementary Figure 8. Additional validation of up-regulated genes.

RT-qPCR was performed on GFP+ FACS-sorted cochlear cells from P3 pups. The level of gene expression in CA-ERBB2 sample is presented as ΔΔCt value (± SD) relative to Control (CTRL) normalized against the expression of the two reference genes, Eef1a1 (E) and Tubb4a (T) (n=3). Significance (*$p<0.05$; ** $p<0.01$; *** $p<0.001$) was determined by unpaired t-test (one-tailed) analysis. Below, UMAP plots and split violin plots showing up-regulated expression of Bglap, Bglap2, Ptgis, Il12a, CD63 and Ank in cells with activated CA-ERBB2.
